# Open Data Are Urgently Needed for One Health-Based Investigations: The Example of the 2024 *Salmonella* Umbilo Multi-Country Outbreak

**DOI:** 10.3390/ijerph22101478

**Published:** 2025-09-25

**Authors:** Alessandra Mazzeo, Celestina Mascolo, Marco Esposito, Lucia Maiuro, Sebastiano Rosati, Elena Sorrentino

**Affiliations:** 1 Department of Agricultural, Environmental and Food Sciences, University of Molise, 86100 Campobasso, Italy; alessandramazzeo@unimol.it (A.M.); maiuro@unimol.it (L.M.); sorrentino@unimol.it (E.S.); 2 General Directorate for Health Protection and Coordination of the Regional Health System-Unit of Prevention and Veterinary Public Health, 80132 Naples, Italy; marco.esposito@regione.campania.it; 3 Department of Agricultural, Forest and Food Sciences, University of Turin, 10124 Turin, Italy

**Keywords:** *Salmonella* Umbilo, multi-country outbreak, buffalo salmonellosis, RASFF alerts, rocket salad, human–animal–environment interface, open data, One Health

## Abstract

In 2024, a significant *Salmonella* Umbilo outbreak was reported across the European Union and beyond, traced to contaminated vegetables originating from the Province of Salerno (Italy). Subsequent on-site inspections in the production area revealed a mismanaged manure storage tank, which became the focus of a GIS-based investigation aimed at locating nearby animal establishments. Within a 1-km radius—encompassing both the tank and the contaminated greenhouses—three buffalo farms were identified. Farm inspections revealed buffalo calves exhibiting enteric symptoms. Fecal samples collected from these animals led to the isolation of *S.* Umbilo genomically linked to the 2024 multi-country outbreak, as well as other serotypes. To thoroughly investigate, data from official EU and Italian databases were analyzed, to detect the presence of *S.* Umbilo in vegetables, buffalo, and other livestock within the Province of Salerno. However, the lack of access to critical data needed to clarify the epidemiological links at the human–animal–environment interface has hindered the full reconstruction of the outbreak dynamics. These limitations underscore the urgent need to implement One Health strategies by promoting interdisciplinary collaboration among veterinarians, physicians, food technologists, biologists and other professionals; leveraging official open access databases; and adopting emerging technologies as interoperable data systems and drone surveillance.

## 1. Introduction

*Salmonella* serotypes differ in host spectrum, virulence, and zoonotic potential. In recent years, there has been an increase in the epidemiological relevance of emerging and previously rare serotypes of non-typhoidal *Salmonella* [1], often implicated in food-borne outbreaks associated with fresh vegetable products and sprouted seeds [2].

However, published official data are presented in an aggregated form [2], and data available in institutional portals lack the national reports that include details about the manufacturing companies and their exact locations [3]. Furthermore, human data are strictly protected. Consequently, elements useful for deeper investigation at the human–animal–environment interface are lacking. Open data, instead, could help highlight additional epidemiological factors that may contribute to these disease outbreaks.

In this context, our work focused on the serious public health alert that emerged between July and December 2024 across the European Union (EU) including Germany, Austria, Denmark, and Italy, in the European Economic Area (EEA) involving Switzerland and the United Kingdom (UK), and beyond, reaching some extra EU countries as Qatar, and the United Arab Emirates. Over 200 human cases of infection and one associated fatality were reported, caused by *Salmonella enterica* subsp. *enterica* serovar Umbilo (*S.* Umbilo) [4]. It is a rare serotype belonging to antigenic group O:28 and presenting the z10 (Phase 1) and e,n,x (Phase 2) H antigens, firstly isolated in Ghana in 1954 from a European woman [5,6].

The 2024 multi-country outbreak was epidemiologically linked to the consumption of rocket salad (RASFF alerts No. 2024-7033) and organic leaf spinach (baby spinach) (RASFF alerts No. 2024-7478) from Italy [4]. Subsequent investigations conducted by Italian authorities identified the production area of the implicated vegetables as the Province of Salerno, in the Campania Region (Italy), an area known for intensive buffalo farming [7].

The ascertained epidemiological link between human infections and fresh vegetables has overlooked the high density of animals in the affected area, which may act as potential shedders of enteric pathogens with zoonotic potential, including serotypes of enhanced pathogenicity. This is particularly important considering that no national programmes are currently in place for the control of salmonellosis in ruminants. Although national programmes for the control of bovine brucellosis and tuberculosis have been reinforced at the regional level in Campania, helping to mitigate the risk of environmental fecal contamination, the presence of salmonellosis in local livestock should still be considered.

In this context, our study sought to elucidate additional hidden factors that may have contributed to the outbreak, other than assessing the potential risk for further salmonellosis outbreaks. To achieve these objectives, we integrated official data generated by veterinary officers responsible for the surveillance of food-borne zoonoses (including some of the co-authors), epidemiological information retrieved from relevant online portals, and evidence drawn from the international scientific literature.

After collecting data on the presence of *S.* Umbilo in the EU and in Italy, the investigation focused on 2024–2025 alerts concerning the presence of *S.* Umbilo and other relevant *Salmonella* spp. serovars in vegetables produced in the Province of Salerno; in livestock reared in the same area; and on the morphological features of the hydrographic canals and manure storage tanks located therein.

## 2. Materials and Methods

### 2.1. Data Collection from Accessible Portals

Data for this study were obtained from the following official database:

- The European Union Centre for Disease Prevention and Control (ECDC) [8].
- The European Union Food Safety Authority (EFSA) [9].
- The European Commission Rapid Alert System for Food and Feed (RASFF) [3].

All the aforementioned EU institutions provide data, atlases, and reports freely accessible online, but only in an aggregate form.

- The National Agriculture Information System (Sistema Informativo Agricolo Nazionale-SIAN) database of the Italian Ministry of Agriculture, Food Sovereignty and Forestry [10].
- The VETINFO portal, the Italian veterinary portal of the Italian Ministry of Health, developed by Istituto Zooprofilattico Sperimentale of Abruzzo and Molise (IZS AM) “Giuseppe Caporale”. This portal also integrates SIMAN–the National Information System for Animal Disease Notification, which is a centralized platform for the mandatory reporting of animal disease outbreaks in Italy, accessible only to authorized veterinary personnel [11].

### 2.2. Field Investigations and On-Site Observations

Field investigations were conducted between November 2024 and January 2025 by local unit of Food Hygiene and Nutrition Service and local Veterinary Unit, supported by regional authorities to assess potential sources of environmental contamination in the area surrounding the greenhouses linked to the multi-country outbreak.

The inspections included the following:

1. Audit of the greenhouse owner focused on:
  - Evaluation of irrigation water quality, with verification of potential microbial contamination.
  - Environmental assessment of the surrounding area through field visits to identify possible sources of contamination, with a focus on drainage channels.
  - Sampling of vegetables and water.
2. Inspection of the area surrounding the greenhouses led to the discovery of an unauthorized and mismanaged manure storage tank, apparently intended for a nearby but unidentified livestock farm. Anticipating this key finding is essential for introducing the subsequent methods.

### 2.3. Georeferencing and Geospatial Mapping

To identify livestock establishment in the area and its related animal species using the storage tank, geographic coordinates of the key site were georeferenced using GPS technology and spatially mapped with Geographic Information System (GIS), version 3.24.0 Tisler (Free Software Foundation, Inc., Boston, MA 02110-1301 United States).

Maps of the study area were generated using QGIS Geographic Information System, version 3.24.0 Tisler (Free Software Foundation, Inc., Boston, MA 02110-1301 USA). The geospatial database included

- GPS coordinates of the rocket salad production site and irrigation channels.
- GPS coordinates of the unauthorized manure storage tank and surrounding wastewater areas.
- Locations of the nearest farms registered in the VETINFO portal, delineating a buffer zone of 1-km radius around the mismanaged manure storage tank.

These data were compiled in Excel spreadsheets, including both geographic coordinates and farm identification codes.

Shapefiles for the study area and irrigation canals were downloaded from the Gistat website [12].

All spatial layers were imported into QGIS, where Excel data were converted to point vector layers for spatial analysis.

### 2.4. Veterinary Fieldwork and Official Microbiological/Genomic Analyses

In the identified farms, veterinary inspections, epidemiological investigations and fecal sample collection from buffalo calves showing enteric symptoms were conducted.

Detection of *Salmonella* spp. was achieved through routine bacteriological and serotyping analyses performed by local official laboratories at the Istituto Zooprofilattico Sperimentale del Mezzogiorno, Portici (Naples, Italy). Where appropriate, genotyping of isolates was carried out by National Reference Centre for *Salmonella* at the Istituto Zooprofilattico Spermentale delle Venezie, Legnaro (Padua, Italy).

## 3. Results

**Figure 1:**
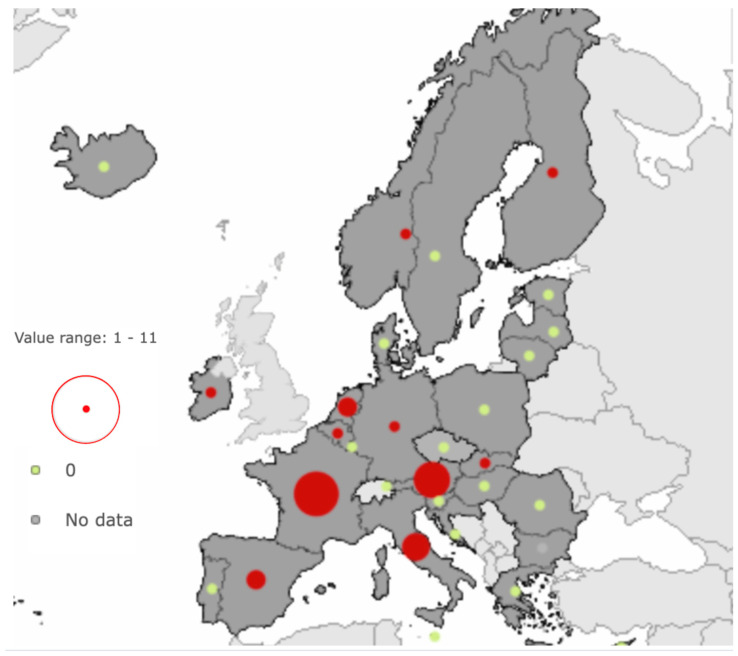
Human cases of *Salmonella* Umbilo infection reported in the EU/EEA in 2023. Countries with reported cases are indicated with a red dot–the larger the dot, the higher the number of cases, with a maximum of 5 in France. Countries with no reported human cases of *S.* Umbilo are marked with a green dot; and countries for which no data are available are marked with a grey dot [13].

*S.* Umbilo has previously been detected only sporadically within the EU and EEA. In 2023, human cases of salmonellosis caused by *S.* Umbilo were reported in Austria, France, Germany, Italy, Slovenia, Spain, and Sweden (Figure 1).

As shown in Table 1, the number of human cases of *S.* Umbilo in the EU/EEA between 2020 and 2023 remained low, with annual reported cases ranging from 15 to 23. The highest number, 23 cases, occurred in 2022. Notably, none of the reported cases during this period resulted in a fatal outcome. France consistently reported the highest incidence, averaging five cases per year. In Italy, three human cases were reported in 2020 (locations not available). In 2021, a single case was documented in the North-West. No cases were recorded in 2022. In 2023, three human cases were identified: one in the North-East and two in the North-West [13].

In contrast to the limited number of human cases of *S.* Umbilo reported in the EU during 2020–2023 (Table 1), a multinational outbreak occurred in 2024, resulting in over 200 confirmed human infections, including one death. As previously reported, the outbreak was linked to the consumption of organic rocket and spinach produced in the Province of Salerno. These vegetables were labeled as Class I, fresh, unprocessed products intended to be consumed raw after washing. In September 2024, Austria submitted a notification to the RASFF portal concerning the presence of *S.* Umbilo in rocket salad (RASFF alert No. 2024.7033). In October 2024, Germany reported the presence of *S.* Umbilo in organic leaf spinach (RASFF alert No. 2024.7478).

Consultation of the RASFF portal revealed that, in the same area, a separate notification was issued regarding the detection of *S.* Livingstone in rocket salad (RASFF alert No. 2024.8024). Additional *Salmonella* serotypes were also reported in vegetables during 2024 and the 1st semester of 2025, though originating from other areas of the Province of Salerno. These include *S.* Napoli (RASFF alert No. 2024.8347) and No. 2025.3978, a frequently detected serotype despite the absence of a clearly identified animal reservoir, and *S.* Stanleyville (RASFF alert No. 2024.8772) (Table 2).

Initial inspections of processing facilities, conducted by local authorities supported by the regional ones, revealed no major hygiene issues. Routinely monitored well water was used for irrigation purposes.

Subsequently, the investigation uncovered a serious irregularity: an unauthorized and mismanaged manure storage tank of unknown ownership was detected, posing a significant risk of overflowing into adjacent irrigation canals when at full capacity. Due to improper management, animal waste from surrounding farms may have entered the drainage systems, potentially introducing microbial pathogens into the agricultural environment.

At the end of 2024, during the epidemiological investigation conducted by veterinarians in the three buffalo farms identified within the 1-km radius surrounding the unauthorized manure storage tank, buffalo calves presenting enteric symptoms were identified. Analyses of the collected fecal samples confirmed salmonellosis outbreaks, which were then registered in the VETINFO portal, involving buffalo calves born between 7 November and 2 December. Four buffalo calves presenting enteric symptoms tested positive for *S.* Senftenberg, antigenic formula 1,3,19:g,[s],t:- (outbreak No. 2024/26) in a livestock hosting 202 buffalo heads. In a second establishment hosting a total of 414 buffalo heads, six buffalo calves showed enteric symptoms and tested positive for *Salmonella* Umbilo (outbreak No. 2025/1). In the third farm, six calves with enteric clinical signs, out of a total of 872 buffaloes, tested positive for *S*. Livingstone infection, antigenic formula 6,7,14:d:l,w (outbreak No. 2025/2) (Table 2) [11]. Notably, two out of the three isolated serotypes had also triggered RASFF alerts in vegetables (Table 2).

Furthermore, RASFF alerts were issued for *S.* Umbilo and *S.* Livingstone in fresh vegetables, supporting the hypothesis of a possible contamination pathway between infected buffaloes and cultivated vegetables, or vice versa. However, no trade ban was reported in RASFF regarding the contamination of fresh vegetables by *S.* Senftenberg (Table 2).

Importantly, none of the aforementioned serotypes were detected in other farmed animal species within the Province of Salerno, namely cattle, swine, sheep, and goats [11].

**Figure 2:**
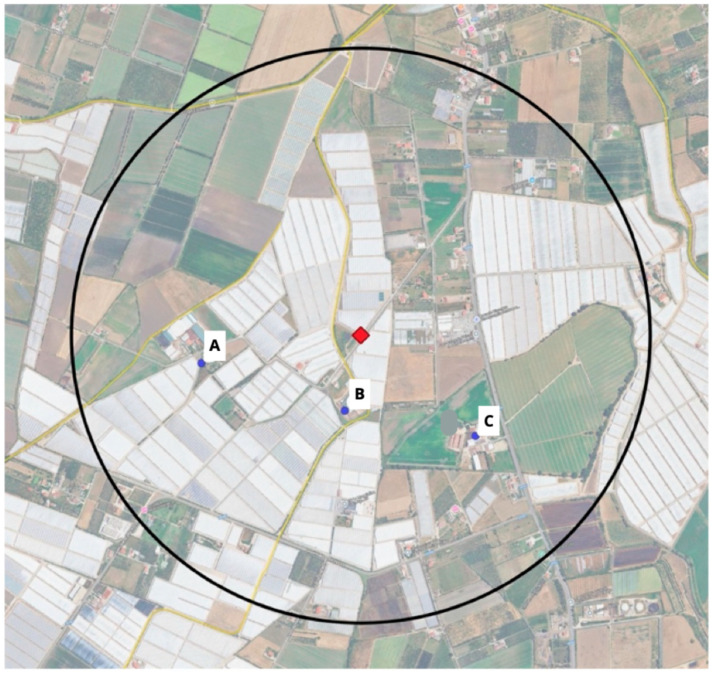
The 1-km radius location of the buffalo establishments (blue dots) presenting *S.* Livingstone (**A**), *S.* Senftenberg (**B**) and *S.* Umbilo (**C**) clinical cases, the manure storage tank (red square), the hydrographic network (yellow lines), and the greenhouses (light areas) from which the contaminated vegetables originated.

Geospatial analysis showed that, within a 1-km radius around the greenhouses associated with the multi-country outbreak (Figure 2, Table 2), were located the following sites:

- The *S.* Umbilo-infected buffalo farm;
- The other two establishments where enteritis in buffalo calves was detected;
- The unauthorized and mismanaged manure storage tank and irrigation channels.

The integration of databank and cadastral data analysis enabled the identification of the property owner, who was also the owner of the buffalo farm affected by *S*. Senftenberg infection.

Core genome Multi-Locus Sequence Typing (cgMLST) was conducted by the relevant national centers to investigate the genetic relationships between *S.* Umbilo isolates from buffaloes and isolates from rocket salad samples and human cases. A close genetic link between the fecal isolates from buffaloes and isolates from the contaminated vegetables was detected, which, in turn, had shown a strong similarity with isolates from human cases, as reported in EpiPulse [4]. The official report was submitted to the Campania Regional authorities (including one of the coauthors of this study) on 13 February 2025.

The two samples collected from the manure storage tank, both from superficial, peripheral areas where manure may have accumulated for a prolonged period and been diluted by rains, tested negative for *Salmonella* spp.

## 4. Discussion

The multi-country outbreak occurred in humans in the EU, EEA, and abroad, caused by vegetables contaminated from *S.* Umbilo [4] and linked to the *S.* Umbilo infection in buffaloes, was confirmed thorough the official test report communicated to the Campania Region authorities and emerged in June 2024 [11], highlighting the ongoing risk posed by enteric pathogens to the local agri-food system.

In Germany, the frequency of isolation of *S.* Umbilo over the previous five years showed a median of three isolates per year, and between 20 and 32 annual human cases were reported by the ECDC for the EU/EEA (including the UK until 2019) over the past ten years (2014–2023) [4].

The Province of Salerno, source of the contaminated vegetables, is also known for its buffalo farms, with 113,421 water buffaloes (*Bubalus bubalis*) raised across 404 farms [11]. This sector represents a valuable economic resource, especially due to its high-quality dairy production, notably the “Mozzarella di Bufala Campana” PDO.

Following on-site inspection and detailed geospatial mapping, the unauthorized and mismanaged manure storage tank was identified as a major risk factor for *Salmonella* transmission. Several infections were detected in buffalo calves during winter 2024, including *S.* Umbilo, *S.* Livingstone, and *S.* Senftenberg, all of which were registered in the VETINFO portal [11]. In particular, mismanagement of the uncovered manure tank, combined with rainfall events, likely caused overflow into adjacent irrigation channels, potentially introducing microbial pathogens into the surrounding environment.

Notably, the delayed collection of water samples from the tank might explain the negative microbiological results, as timeliness in sampling is a key factor in the reliable microbiological detection, as environmental factors such as temperature and stratification of materials play a critical role in *Salmonella* survival [14].

The presence of enteric pathogens such as *S. enterica*, transmissible via the fecal-oral route, having zoonotic potential via direct and indirect contact including foods, and capable of persisting in the environment through biofilm formation, poses a tangible risk [15]. Even greenhouse cultivation of vegetables does not eliminate the risk of contamination via irrigation water or drainage overflow.

This scenario overlaps the need to combat bovine brucellosis (now brought to zero cases), and bovine tuberculosis, which have long affected livestock, resulting in a non-Disease-Free Status for both diseases. Consequently, regional regulations have become more stringent, placing particular emphasis on controlling environmental contamination and adopting a One Health approach [16,17,18]. The adopted measures span the zootechnical, agricultural and environmental sectors, and include restrictions such as the prohibition of green fodder, surveillance of irrigation and drainage systems, and stringent livestock waste management protocols, with enforcement through penalties for non-compliance [7].

Historically, *S.* Umbilo has been sporadically detected in Italy. In 2004, it was identified in a sheep during a surveillance investigation conducted at a port [19]. Between 2016 and 2022, *S.* Umbilo was detected in wildlife in the Emilia Romagna Region, but only in a single badger out of 927 tested animals [20]. In Campania, the serotype was reported in buffaloes and wild boars between 2011 and 2021, with isolation from organs of four buffaloes out of 120 tested and two wild boars out of 82 tested animals [21]. A 2008–2009 study analyzing the intestinal contents of 248 buffalo calves affected by gastroenteritis with a lethal outcome revealed the presence of *S.* Umbilo in two animals [22]. Notably, no outbreaks have been registered in buffaloes from the Province of Salerno since 2010, as noted extending our study at VETINFO-SIMAN data concerning the last fifteen years. These limited data underscore the urgent need for further investigations to determine the extent of its presence across the country and to assess the potential risk to consumers. In past cases in the UK, *S.* Umbilo was previously detected (2001) in samples of organic rocket originating from Italy; in that instance, the authors hypothesized transmission via lizards [23].

The detection of other *Salmonella* spp. serovars (*S.* Livingstone and *S.* Senftenberg) in buffalo farms raises further concern due to their zoonotic potential [24,25,26,27,28]. These findings underscore buffalo farming as a high-risk sector requiring rigorous and continuous zoonotic prevention measures [7,29]. This is particularly important, because contaminated environments foster microbial proliferation, thereby facilitating the spread of antibiotic resistance. Resistance genes can be transmitted vertically and horizontally via mobile genetic elements, quorum sensing, and other mechanisms, increasing the persistence and zoonotic potential of strains [30,31,32].

Antibiotic resistance remains a major challenge in managing foodborne *Salmonella* infections. Multiresistant strains, particularly *S. enterica* serotypes Typhimurium, Enteritidis, and Infantis, have been isolated from chicken meat, eggs, pork, and cured meat [30]. Of particular concern is the presence of mobile megaplasmids, such as the pESI plasmid, which can carry multiple resistance genes and virulence determinants, increasing zoonotic potential and persistence of *Salmonella* along the food chain; in poultry strains from Korea, the pESI plasmid was associated with a multiresistance rate of 99.2% [30]. This issue extends to other Gram-negative bacteria involved in enteric diseases in calves, including *Escherichia coli*, *Klebsiella pneumoniae*, and *Proteus* spp., which also harbor combined virulence and resistance genes, highlighting their potential role in the dissemination of antimicrobial resistance [31,32].

The cultivation of raw-consumed vegetables in areas of high livestock density, such as Salerno, requires particular attention. For example, *Rucola della Piana del Sele* (*Diplotaxis tenuifolia*, *L.*), Protected Geographical Indication (PGI)*,* cultivated in the Province of Salerno, is monitored under the “Goccia Verde” irrigation certification.

In Italy, similar vigilance is required in the Po Valley, where intensive livestock production overlaps with horticultural PGI crops. The presence of *S.* Dublin in cattle deserves particular attention, as this serovar is host-adapted, cattle-associated, and, while only sporadically causing disease in humans, has shown increasing incidence, antimicrobial resistance, and clinical severity in recent years. Multidrug-resistant (MDR) strains are frequently isolated in veterinary settings, particularly in beef calves and dairy cattle [33,34,35,36]. Infections with *S.* Dublin are often associated with higher rates of bloodstream infections, hospitalization, and mortality compared to other *S. enterica* serotypes. Importantly, clinically asymptomatic animals, including bulls, can shed the bacterium, which may persist in the reproductive tract [33,34,35,36].

Following the isolation of *Salmonella* Umbilo, *S*. Livingstone, and *S.* Senftenberg in buffalo farms, in the Province of Salerno targeted measures were implemented to address the outbreaks and prevent the spread of the epidemic among livestock, horticultural products, and humans. A Health Management and Control Plan (PSG) for dairy farms, integrating biosecurity and monitoring protocols, was drafted and implemented for a six-month period, in compliance with the protocol elaborated by the National Reference Centre (NRC) for *Salmonella*, which also elaborates the National Control Plan for Salmonellosis in Poultry [37,38].

Our study highlights the urgent need to foster meaningful engagement among stakeholders in the livestock, dairy, and vegetables production sectors, raising awareness of the interconnectedness of supply chain components and the vulnerability of their primary income sources. Effective communication initiatives are essential, targeting both operators and the general public, to ensure shared responsibility in sustainable land and resource use. Emphasis should be placed on proper disposal of antibiotics used at the household level for infectious diseases as salmonellosis in young animals, to counteract the increasing antimicrobial resistance of *Salmonella* spp. serotypes worldwide. Identified threats should be systematically analyzed to enable proactive interventions linking human health with agricultural and livestock practices [39], including revision and adaptation of Good Manufacturing Practice (GMP) and company-specific HACCP plans.

Interprofessional collaboration at the human–animal–environment interface is crucial for implementing the One Health approach. Environmental monitoring can be enhanced through innovative monitoring technologies, such as drone surveillance under privacy-compliant frameworks, allowing verification of farm hygiene, waste disposal, and drainage management, while supporting rapid intervention when needed.

A critical challenge identified in our study is the restricted accessibility of comprehensive epidemiological and microbiological data. Key datasets that are currently available in open access platforms, covering human cases (including potential occupational infections), associated clinical complications, antimicrobial resistance profiles, and genomic analyses of isolates from animals, food, feed, and environmental samples, are insufficient. This lack of comprehensive open access data severely limits the ability to reconstruct events leading to public health emergencies, to understand epidemiological links across sectors, and to align and compare findings obtained by different laboratories and institutions.

In our opinion, open access to these datasets is therefore essential to enable rigorous interdisciplinary research, to facilitate timely and effective public health interventions, and to strengthen the practical implementation of the One Health approach. Moreover, providing researchers, including academic students at various levels, with access to these data is crucial for modern education and training. It allows students to acquire up-to-date information, critically analyze and connect it to previous knowledge, and develop the interdisciplinary skills required for their future professional practice. Structured training around the principles of One Health ensures that these approaches become an integral and instinctive part of professional decision-making.

Ultimately, broader access to epidemiological and microbiological data could greatly improve the speed and effectiveness of outbreak investigations, enabling the rapid identification of the origin of epidemics and immediate interruption of transmission chains, thereby protecting human, animal, and environmental health.

## 5. Conclusions

Our work represents a milestone in epidemiological investigations at the human–animal–environment interface, as it strongly advocates for the creation of open access databanks to support cross-sectoral research, an essential step toward effectively addressing public health emergencies that would otherwise remain unresolved. It highlights the critical limitations imposed by the absence of open access data, which are indispensable for comprehensive analyses of public health events. Moreover, it emphasizes the need for the development of advanced informatic tools designed to integrate heterogeneous databases from distinct sectors, which are conventionally considered isolated but are interconnected through the ability of pathogens to cross artificial human-imposed boundaries.

## Figures and Tables

**Table 1:** Number of human cases of *Salmonella* Umbilo infection reported in the European Union and in the European Economic Area (EU/EEA) in the period 2020–2023 [13].

EU/EEA MEMBER STATES	2020	2021	2022	2023
	No. of Human Cases	No. of Human Cases	No. of Human Cases	No. of Human Cases
AUSTRIA	4	0	2	4
BELGIUM	1	1	0	1
CROATIA	0	0	1	0
CZECHIA	0	1	0	0
FINLAND	1	0	0	1
FRANCE	5	5	5	5
GERMANY	0	2	3	1
IRELAND	1	0	0	1
ITALY	3	1	0	3
MALTA	0	0	1	0
NETHERLANDS	2	1	2	2
NORWAY	1	0	0	1
SLOVAKIA	1	0	0	1
SLOVENIA	0	0	4	0
SPAIN	0	4	0	0
SWEDEN	0	0	5	0
TOTAL	19	15	23	20

**Table 2:** *Salmonella* spp. serotypes detected in buffaloes and/or in rocket salad and organic leaf spinach in the Province of Salerno in 2024 and in the 1st semester of 2025.

*Salmonella* Serotypes	Infection in Buffaloes	RASSF Alerts	Location of *Salmonella*-Positive Greenhouses and/or Buffalo Farms in Province of Salerno
*S.* Umbilo	YES	RASFF Alert No. 2024.7033 ROCKET SALAD related to the 2024 multi-country outbreak	EBOLI Site of origin of the multi-country outbreak 2024
*S.* Umbilo	YES	RASFF Alert No. 2024.7478 LEAF SPINACH related to the 2024 multi-country outbreak	EBOLI Site of origin of the multi-country outbreak 2024
*S.* Livingstone	YES	RASFF Alert No. 2024.8024 ROCKET SALAD not related to human outbreaks	EBOLI Site of origin of the multi-country outbreak 2024
*S.* Senftenberg	YES	NO RASFF ALERT	EBOLI Site of origin of the multi-country outbreak 2024
*S.* Napoli	*Not included in the epidemiological investigation*	RASFF Alert No. 2024.8347 ROCKET SALAD	BATTIPAGLIA and PONTECAGNANO FAIANO
*S.* Stanleyville	*Not included in the epidemiological investigation*	RASFF Alert No. 2024.8772 ROCKET SALAD	PONTECAGNANO FAIANO
*S.* Napoli	*Not included in the epidemiological investigation*	RASFF Alert No. 2025.3978 ROCKET SALAD	BELLIZZI

## Data Availability

The data presented in this study are available on request from the corresponding author.
